# A Comparison of Antibacterial Properties of Tachyplesin, Thanatin, and Enterocin P on *Enterococcus faecalis*

**DOI:** 10.14744/eej.2021.04696

**Published:** 2022-03-15

**Authors:** Armita ROUHANI, Afshin JAVADZADEH, Abbas TANHAEIAN, Sara NAVABI

**Affiliations:** From the Department of Endodontics (A.R.), Mashhad University of Medical Sciences, Faculty of Dentistry, Mashhad, Iran; Department of Endodontics (A.J.), Private Practice, Mashhad, Iran; Department of Biotechnology and Plant Breeding (A.T.), Ferdowsi University of Mashhad, Faculty of Agriculture, Mashhad, Iran; Department of Endodontics (S.N. 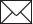 snavabi2000@yahoo.com), Dental Research Center, Mashhad University of Medical Sciences, Mashhad, Iran

**Keywords:** Antimicrobial peptide, endodontic re-treatment, Enterocin P, *Enterococcus faecalis*

## Abstract

**Objective:**

*Enterococcus faecalis (E. faecalis)* is one of the persistent microorganisms responsible for the failure of root canal treatments. This study investigated the antibacterial property, the time-killing of 3 peptides, namely Tachyplesin, Thanatin, and Enterocin P, on *E. faecalis*.

**Methods:**

In this study, recombinant peptides were synthesized via secretory synthesis. The peptides were then purified and isolated using affinity chromatography after which their purification was evaluated through SDS-PAGE. The antimicrobial activity of peptides against *E. faecalis* was tested using the minimum inhibitory concentration test (MIC), the minimum bactericidal concentration test (MBC), and the time-killing assay.

**Results:**

Based on antimicrobial tests, a similar value was observed for the MIC and MBC in the recombinant peptide of Enterocin P. The concentration of MBC was twice as much as that of MIC for Tanatin and Tachyplesin. The time-killing-assay antimicrobial test showed that Enterocin P has a better pattern and antimicrobial activity than the other two peptides; all three peptides have weaker antimicrobial activities than sodium hypochlorite.

**Conclusion:**

Considering the equivalence of MIC and MBC in the recombinant peptide of Enterocin P, it can be a viable replacement for traditional disinfectants and medicaments used in root canal treatment procedures.

**HIGHLIGHTS**

• Despite its high success rate, endodontic treatment might still fail due to the persistence of microorganisms in the root canal system.
• In spite of cleaning and shaping of the root canal system, *Enterococcus faecalis* (*E. faecalis*) can survive in the canal as a single strain.
• Traditional intracanal disinfectants and dressings such as sodium hypochlorite (NaOCl), chlorhexidine (CHX), and calcium hydroxide might not have all the desirable properties to eliminate persistent microorganisms such as *E. faecalis*.
• Considering the importance of the bactericidal effects of antibacterial agents in root canal treatment success, Enterocin P has the potential to be a better choice as an intracanal medicament for endodontic retreatment in comparison to Thanatin and Tachyplesin. However, this finding needs to be confirmed by further studies.
• Antimicrobial peptides can be a potential intracanal medication due to the time-killing assay test results and their antimicrobial activity.

## INTRODUCTION

The main cause of dental pulp infection is the existence of carious lesions. The rate at which such infections progress is determined by the virulence and proliferation rate of microorganisms (1). These microorganisms cause pulpitis which requires root canal treatment. Root canal treatment is considered a fairly predictable procedure with a success rate of 86 to 98%. Its success is determined by lack of pain, inflammation, sinus tracts, and the presence of normal periapical bone and lamina dura (2). Despite its high success rate, root canal treatment might still fail due to the persistence of microorganisms in the root canal system that could be a result of improper mechanical and chemical disinfection or the microorganisms’ evasion (in dentinal tubules), (3, 4).

One such microorganism is *Enterococcus faecalis* (*E. faecalis*), which is a non-motile, gram-positive, facultative anaerobic with a size of 0.6-2.0 μm. It resists a wide variety of difficult growth conditions, including temperatures between 10 and 45°C, as well as hypotonic, hypertonic, acidic, or alkaline environments (5). The prevalence of *E. faecalis* in primary endodontic infections is much less than that of persistent endodontic infections which have been reported to be 24% to 77% in previously treated teeth with peri-radicular lesions (6). It can adhere to host cells, compete with other bacterial cells, and alter host responses. It can also suppress lymphocytes which could lead to root canal treatment failure. Furthermore, it can reach the root canal during or after treatment or between sessions (6).

None of the traditional intracanal disinfectants and dressings such as sodium hypochlorite (NaOCl), chlorhexidine (CHX), and calcium hydroxide (CH) have all the desirable properties to eliminate all the microorganisms including *E. faecalis* (7, 8). NaOCl is an effective irrigant for *E. faecalis*, even in biofilms, but it must be used very carefully to avoid contact with the oral tissue (9). CH is ineffective against *E. faecalis* in the root canal system since it can hardly penetrate dentinal tubules that *E. faecalis* resides in (10). The effectiveness of 2% CHX, (gel or liquid formulation) in eliminating *E. faecalis* from the root canal space and dentinal tubules is limited by its inability to dissolve the biofilm created by microorganisms and by its toxicity to vital tissues in high concentrations (11).

Anti Microbial Peptides (AMPs) are endogenous biomolecules that act by interacting with the surface of microbial membranes or acting inside the target cell, leading to their broad activity spectrum (12). Tachyplesin, Thanatin, and Enterocin P are three AMPs that display a net positive charge (due to the presence of lysine, arginine, and histidine). They are also amphipathic and the hydrophobic parts play a role in membrane permeabilization (13, 14). Their low antimicrobial concentration and tissue repair capability make them candidates for use as an intracanal medicament in cases of endodontic failure (12). Furthermore, they have been shown to have no toxic side effects (15). Considering the increasing resistance of bacteria to antibiotics, in addition to the shortcomings of traditional disinfectants against *E. faecalis*, searching for alternatives including antimicrobial peptides (AMPs) seems a reasonable choice (13). 

The purpose of this study was to determine the potential application of Tachyplesin, Thanatin, and Enterocin P as a root canal medicament by evaluating their antibacterial activity on *E. faecalis* using minimum inhibitory concentration (MIC), minimum bactericidal concentration (MBC), and time-kill tests.

## MATERIALS AND METHODS

The present study was carried out at Mashhad University of Medical Sciences where the effects of Thanatin, Thachyplesin, and Enterocin P on *E. faecalis* were studied. Amino acid sequences and information of the peptides obtained from the Antimicrobial Peptide Database (APD) are presented in Table 1.

**TABLE 1.** Amino acid sequences and information of the peptides obtained from Antimicrobial Peptide Database (APD)

Name/Class	Thanatin	Tachyplesin 1	Enterocin P
APD ID	AP00102	AP00214	AP00853
Source	Podisus Maculiventris	Tachypleus Tridentatus	Enterococcus Faecium P13
Sequence	GSKKPVPIIYCNRRTGKCQRM	KWCFRVCYRGICYRRCR	ATRSYGNGVYCNNSKCWVNW
			GEAKENIAGIVISGWASGLAGMGH
Length	21	17	44
Net charge:	6	7	1
Hydrophobic residue %	0.28	47	40
Boman Index (Kcal/mol)	2.81	3.53	0.76
3D Structure	BETA	BETA	Unknown
Activity	Anti-Gram+ & Gram-, Antifungul	Anti-Gram+ & Gram-, Antiviral, Anti-HIV	Anti-Gram+

### The recombinant peptide synthesis

For cloning and expressing the peptide genes, human embryonic kidney cells (HEK293) (Pasteur Insititute, Tehran, Iran) were used. According to the secretion signal and coding sequence of the recombinant peptides in the vector, the transfected cell lines had the capability of synthesizing and secreting the peptides into the medium. The transfected cell lines were then harvested, and the medium containing the recombinant peptide was collected for the antimicrobial assay.

The transfected human embryonic kidney cells (HEK293) were cultured in the Dulbecco Modified Eagle Medium (Sigma-Aldrich Co. LLC, St. Louis, USA) containing 10% fetal calf serum (GIBCO Laboratories, Life Technologies, Inc., New York, USA) - inactivated by heat and a combination of penicillin, streptomycin (GIBCO Laboratories, Life Technologies, Inc., New York, USA) - and 2 millimoles of glutamine (Biosera, Ringmer Lewes, England), cultivated in an incubator with 5% Co_2_, and 85% of humidity, at 37°C. The cell's passage occurred every 2-3 days at 0.2×10^6^ cell/ml of the cell concentration. After each passage, the culture containing the recombinant peptide was collected from the transfected cells.

### Purification and measurement of the peptide concentration

In this phase, dry resin (His-Select Nickel Affinity Gel, Sigma; 1 mL) was washed on an Econo-Pac column (Chromatography Columns; BIO-RAD) with 5 vol of water and 10 vol of buffer A (50mM NaH_2_PO_4_, 300 mM NaCl, 10 mM Imidazole (Sigma®); pH 8.0) and then incubated for 15 minutes at 4°C. Following this, the column was equilibrated with 10 vol of buffer A and was then re-incubated for a further 15 min at 4°C. 

Having centrifuged the column, each peptide’s supernatant was diluted two-fold in an equal volume of buffer B (100mM NaH_2_PO_4_, 600mMNaCl, 20 mM imidazole at pH 8.0). 1 mL of resin was added, and after overnight incubation at 4°C, the mix was loaded onto the chromatography column and washed twice with buffer A.

Three milliliters of buffer C (50 mM NaH_2_PO_4_, 300 mM NaCl, 250 mM imidazole (Sigma®); pH 8.0) was added to the mixture and washed three times. Each wash was concentrated nine to tenfold by ultrafiltration (to reach a final volume of of1 mL). Finally, it was dialyzed against PBS buffer overnight at 4°C (Slide-A-Lyser® Dialysisassette 0.5-3 mL/3500 Da, Pierce®). Protein concentration was measured by the Bradford method (BioRad Protein Assay) according to the manufacturer’s instructions, using BSA (Protein Assay Standard II) as a standard. After each concentration or purification step, the protein fractions (peptides) were assessed qualitatively by SDS-PAGE. 

### The antimicrobial assay

In order to evaluate the antibacterial effectiveness of the peptides, strains of *Enterococcus Faecalis* (*E. Faecalis*) ATCC# 29121 (Pasteur Institute, Tehran, Iran) were obtained from a research organization. A bacterial suspension containing about 1.5×10^8^ CFU/ml was prepared by culturing the bacteria on anaerobe blood agar plates incubated at 37°C for 24 hours and adjusting it to the turbidity of a 0.5 McFarland standard. The minimum inhibitory concentration (MIC), Time-killing assay, and the minimum bactericidal concentration (MBC) were also determined by a broth microdilution test.

### The Minimum Inhibitory Concentration (MIC) analysis

As for this analysis, the bacterial suspension was exposed to a dilution series of the peptides using a micro broth dilution method in 96-well microplates for 24 hours (three times for each peptide). The minimum dilution of the peptides leading to the complete growth inhibition (the undetectable growth in the media) and the bacterial growth was then measured by the change to the optical density (turbidity) of the wells in the microplate.

Regarding the MIC analysis, 95μL of the Mueller Hinton liquid (Merk, Darmstadt, Germany) was first added to each well of the 96-well microtiter plates. 5 μL of the bacterial suspension, adjusted to the turbidity of a 0.5 McFarland standard, was added to each well. 100 μL of the peptide with its highest concentration was added to the first well. Other wells were also filled with lower concentrations up to the 10^th^ well. Well number 11 only contained the medium with no bacteria and was considered as the negative control. Well number 12 was considered as the positive control group that contained the medium and the bacterial suspension with no AMP. The plates were then incubated at 37°C for 18-24 hours. Following the termination of the incubation period, the well turbidity was inspected visually. The first well in the series with no sign of visible bacterial growth was considered as MIC. 

### The Minimum Bactericidal Concentration (MBC)

MBC was determined using the Broth tube dilution method by culturing the contents of the wells with no sign of bacterial growth. The tubes were incubated for 24 hours at 37°C in an incubator. The MBC was defined as the lowest concentration in which no bacterial growth was detectable.

### Time-kill assay

Three tubes of nutrient culture medium containing a bacterial suspension of 5×10^5^ CFU/ml were used for each peptide and sodium hypochlorite (for comparison). Concentrations equal to MIC, two, and four times of the MIC of each peptide, were then prepared. A suspension of the bacteria (5.0×10^5^ CFU/mL) was added to each tube and then incubated at 37°C. 

Bacteria were harvested at time intervals of 0, 5, 10, and 30 minutes, and 1, 16, 24, and 36 hours. 10 μL of each dilution was spread on BHI agar plates. After overnight incubation, the number of colony-forming units (CFUs) was counted. The plates in which a 90% cell death at 6 hours and 99.99% at 24 hours was observed were the ones with no colony growth (negative). Other plates, however, had shown colony growth (positive). 

These procedures were employed in triplicates.

### Statistical analysis

The data of the present study were analyzed using the SPSS statistical software program (version 18.0, IBM, Chicago, IL, USA).

## RESULTS

Peptide concentrations subsequent to purification were 886.4 µg /ml, 767.92 µg /ml, and 447 µg /ml for Enterocin P, Tachyplecin, and Thanatin, respectively. SDS gel was used for ensuring the quality of the peptide purification. Tachyplesin and Enterocin P bands were observed at 3 and 5.4 KD respectively. Thanatin’s synthesis was validated using a well with cultured transfected cells alongside a culture well with no transfected cells (to ensure the size of the Thanatin peptide, insulin was run alongside it. Results of the SDS-PAGE analysis showed that the peptides were properly produced in transfected HEK cells with an appropriate secretion rate in the culture. 

**Figure 1.**
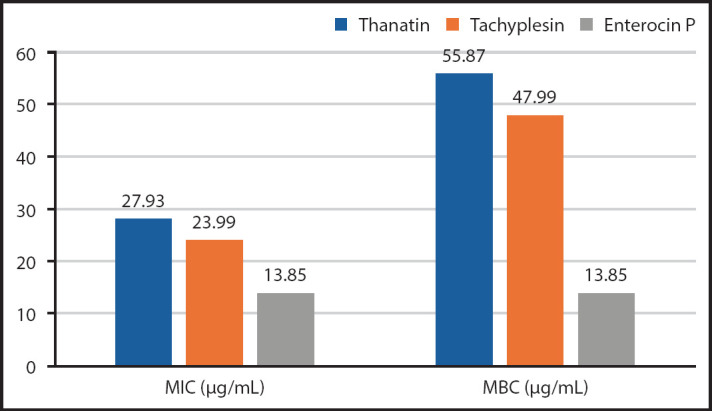
The MIC and MBC values for all three peptides against the assessed bacteria MIC: Minimum inhibitory concentration test, MBC: Minimum bactericidal concentration test

The MIC and MBC values for all three peptides against the assessed bacteria have been presented in Figure 1. According to the results obtained, the MIC and MBC number was the smallest for Enterocin P, while for Thanatin it was the largest. MIC and MBC have the same value for Enterocin P. Enterocin P was found to be of the strongest effects on *E. faecalis*, inhibiting the bacterial growth and killing bacteria at 13.85 μg/mL. Besides, Thanatin demonstrated about 4 times weaker bactericidal effects (55.87 μg/mL) than Enterocin P (13.85 μg/mL) on *E. faecalis*. However, its bacteriostatic effect (27.93μg/mL) was nearly half of Enterocin P (13.85μg/mL). Tachyplesin’s bactericidal and bacteriostatic effects on *E. faecalis* were comparable to Thanatin’s.

The time-killing assay results have been presented in Table 2. At MIC concentration, Enterocin P acted at a faster rate than the other two peptides, but at 2*MIC and 4*MIC, they acted at the same rate. All three peptide's antimicrobial activities were slower than that of sodium hypochlorite.

**TABLE 2.** Time-killing assay results of antibacterial agents at 6 different times

Antibacterial agent	Concentration (µg/mL)		5 minutes	10 minutes	30 minutes	1 hour	16 hours	24 hours	36 hours
Thanatin	MIC	27.93	+	+	+	+	+	+	+
	2*MIC	55.87	+	+	+	+	-*	-	-
	4*MIC	111.74	+	+	+	+	-	-	-
Tachyplesin	MIC	23.99	+	+	+	+	+	+	-
	2*MIC	47.99	+	+	+	+	-	-	-
	4*MIC	95.98	+	+	+	+	-	-	-
Enterocin P	MIC	13.85	+	+	+	+	-	-	-
	2*MIC	27.7	+	+	+	+	-	-	-
	4*MIC	55.4	+	+	+	+	-	-	-
Sodium Hypochlorite	MIC	13.58	+	-	-	-	-	-	-
	2*MIC	27.7	-	-	-	-	-	-	-
	4*MIC	55.4	-	-	-	-	-	-	-

*Negative: The plates in which a 90% cell death at 6 hours and 99.99% at 24 hours is observed, MIC: Minimum inhibitory concentration test

## DISCUSSION

Antimicrobial peptides (AMPs) are natural agents against bacterial infection. They have a nonspecific mode of action based on membrane disruption, making them less likely to induce bacterial resistance. The effects of some AMPs on various bacteria have been previously studied (16). AMPs can prevent and eliminate existing biofilms by specifically targeting bacterial biofilms (17). Furthermore, AMPs have also been studied as potential new anticancer drugs which mainly have cytotoxic activity against cancer cells and unhealthy cells (18). The pediocin-like bacteriocin, termed Enterocin P (EntP), has a broad-spectrum antimicrobial effect on spoilage bacteria such as *E. faecalis* (19). Its antimicrobial mechanism is making specific holes in the cytoplasmic membranes of bacteria. These potassium ion-conducting pores impair the electrochemical transmembrane potential through the accumulation of potassium ions inside the cells (20). Tanhaeeian et al. (21) had previously demonstrated that the EnP recombinant peptide had an effective antibacterial effect on *streptococcus mutans*, *streptococcus salivarius, streptococcus oralis*, and *Enterococcus faecalis* with the MIC values of 3-13 μg/ml. Enterocin P is stable at very low and high temperatures and tolerant in a pH of 2-11. Its ability to withstand acidic and neutral pH justifies its application in the acidic environment of the oral cavity (19).

Tachyplesin is a class of AMPs derived from the hemocytes of the horseshoe crab (Tachypleus tridentatus). It inhibits the growth of both gram-negative and gram-positive bacteria at low concentrations and forms a complex with bacterial lipopolysaccharide (22). Thanatin is derived from the hemipteran insect *Podisus maculiventris* and its antimicrobial effect on *E. coli, Salmonella typhimurium, Klebsiella pneumoniae*, and *Enterobacter cloacae* with MICs <1.5 μM has been reported in several research studies (23, 24). To the best of our knowledge, studies concerning the effects of Tachyplesin and Thanatin on root canal *E. faecalis* are rare. Based on the results of our study, although these two peptides do not have bacteriostatic and bactericidal effects as much as Enterocin P does, it is quite possible to consider them as intracanal medicaments (with concentrations higher than that of Enterocin P).

*E. faecalis* is found 9 times less in root canals of teeth with primary endodontic infections than those previously treated (25). Current technology and limitations of traditional disinfectants can be the reason for this high rate of post-endodontic treatment infection by resistant pathogens including *E. faecalis* (6). *E. faecalis* is persistent due to its survival and virulence factors including, adhesion substance (AS), surface adhesins, sex pheromones, lipoteichoic acid, extracellular superoxide products, lytic enzymes, angiotensin-converting enzymes (ACE), and serine protease (Spr) helping it in binding to the tooth structure (26). We chose to study the bactericidal and bacteriostatic effects of three AMPs on *E. faecalis* due to its role in persistent endodontic infections and endodontic treatment failure (27). In a systematic review by Alghamdi et al. (28), most studies mentioned E.faecalis as the primary pathogen associated with root canal treatment, and which has characteristic properties that make it capable of escaping disinfection chemicals.

According to the results of this study, Enterocin P had the strongest bacteriostatic and bactericidal effect on *E. faecalis* with an equal MIC and MBC of 13.85 μg/mL. This equality proves the high antimicrobial quality of this peptide. Thanatin and Tachyplesin’s MBC was nearly twice their MIC. Considering the importance of the bactericidal effect of the antibacterial agent in endodontic treatment success, Enterocin P has the potential to be a better choice as an intracanal medicament for endodontic retreatment in comparison to Thanatin and Tachyplesin. Although this finding needs to be confirmed by further studies including statistical analysis. Although all three peptides showed a slow antimicrobial effect based on the time-killing assay, Enterocin P proved to be faster while using the MIC concentration (99.99% cell death at 16 hours) than Tachyplesin (36 hours) and Thanatin (+36 hours). However, at 2*MIC and 4*MIC concentrations, all three peptides caused 99.99% cell death at 16 hours. Based on the slow action of these peptides, their use as disinfectant irrigants is not likely unless at high concentrations (higher than 4*MIC, 99.99% cell death happens through 16 hours), but their bactericidal effect makes them an interesting choice as intracanal medicaments.

Calcium hydroxide is one of the most commonly used intracanal (29) medicaments which has desirable properties such as promoting healing and repair, stimulating fibroblasts, and as a remedy for internal resorption. However, it has some disadvantages including its association with primary tooth resorption, dissolution with time, degradation during tooth flexure, and marginal failure with amalgam condensation; it does not adhere to dentine or resin restorations (29). Moreover, the antibacterial effect of calcium hydroxide requires direct contact with microorganisms (30), and it often fails to eliminate bacteria residing deep within the dentinal tubules (31). *Enterococcus faecalis* is one of the known bacteria that can invade dentinal tubules and survive chemical and mechanical counteractions taken against it (32). It has even been reported that *Enterococcus faecalis* can resist calcium hydroxide intracanal dressing for more than 10 days (10). According to the present study, antimicrobial peptides have proved to be considered as a potential intracanal medication due to the time-killing assay test results and their antimicrobial activity. Therefore, these peptides can be introduced as valid alternatives to common intracanal medicaments such as calcium hydroxide. Nevertheless, further controlled studies are recommended to evaluate the antimicrobial properties of such peptides against *E. faecalis* cultured in root canal treated teeth.

## CONCLUSION

Considering the equivalence of MIC and MBC in the recombinant peptide of Enterocin P, this bacteriocin can be considered as an intracanal medicament to eliminate *E. faecalis* from the root canal. Further studies are still required on the dose-dependent antibacterial effects of AMPs and *in-vivo* studies to determine proper delivery methods and ensure the absence of secondary symptoms after their administration to the patients.
